# The cellular receptor for enterovirus 71

**DOI:** 10.1007/s13238-014-0092-6

**Published:** 2014-08-08

**Authors:** Yue Liu, Michael G. Rossmann

**Affiliations:** Department of Biological Sciences, Purdue University, West Lafayette, IN 47907 USA

The *Picornaviridae* family consists of a large group of non-enveloped, icosahedral viruses with a single, positive-stranded RNA genome. The family includes pathogens that are notable for a variety of human and animal diseases (Racaniello, 2007). Picornaviruses initiate infections by attachment to host cell receptor molecules and then undergo uncoating to release the viral genome into the cytosol for replication (Racaniello, 2007). The enterovirus (EV) genus is perhaps the most intensively studied genus among the 26 known genera.

X-ray crystallography (Hogle et al., 1985; Rossmann et al., 1985), cryo-electron microscopy (cryo-EM) (Olson et al., 1993; Bubeck et al., 2005b) and molecular biology have unraveled some of the molecular mechanisms of EV cell entry (Tuthill et al., 2010). Specifically, a canyon-like cleft on the surface of the virus encircling each pentameric vertex is frequently the binding site for putative cellular receptors whose structure belongs to the immunoglobulin superfamily (Table 1) (Rossmann et al., 2002). When these receptor molecules bind into the “canyon” of the EVs, the “pocket factor”, a fatty acid-like molecule residing in a hydrophobic pocket underneath the “canyon”, is ejected (Rossmann, 1994; Xiao et al., 2005; Xiao et al., 2011). This destabilizes the virus and triggers the uncoating process leading to the formation of uncoating intermediate A particles (Rossmann et al., 2002; Bubeck et al., 2005a; Zhang et al., 2008). When a receptor binds into the canyon, the base of the canyon is depressed towards the center of the virus thus diminishing the size of the pocket that contains the pocket factor, causing the stabilizing pocket factor to be ejected. This happens because the peptides forming the base of the canyon also form the roof of the pocket. Thus, in essence, either the receptor or the pocket factor can bind to the virus, but not both (Smith et al., 1986; Rossmann, 1989). However, receptors to some EVs, such as the minor group rhinoviruses, bind to other locations on the viral surface and don’t assist in viral uncoating (Table 1) (Plevka et al., 2010; Tuthill et al., 2010; Shakeel et al., 2013).

**Table 1:** Selected enteroviruses and their cell entry characteristics

Virus	Receptor	Receptor structural feature	Binding site on virus	Initiation of uncoating
Poliovirus 1–3	CD155	Ig-like	Canyon	Yes
Coxsackievirus A21	ICAM-1	Ig-like	Canyon	Yes
Major group rhinoviruses	ICAM-1	Ig-like	Canyon	Yes
Coxsackievirus B3	CAR	Ig-like	Canyon	Yes
Coxsackievirus A9	Integrin αvβ6, αvβ3	V-shaped, 12 domains	Near two-fold axes	No
Echovirus 7	CD55	Four short consensus repeats	Near two-fold axes	No
Minor group rhinoviruses	LDLR family	“arc”-like arrangement of ligand binding domain repeats	Shallow pits near five-fold axes	No

The table is based on observations by Rossmann et al., (2002), Plevka et al., (2010), Tuthill et al., (2010) and Shakeel et al., (2013)

*ICAM-1* intercellular adhension molecule 1, *CAR* coxsackievirus and adenovirus receptor, *LDLR* low density lipoprotein receptor

Human enterovirus 71 (EV71) is currently a major causative agent for hand, foot and mouth disease with occasionally severe neurological complications. A growing number of EV71 outbreaks have been reported in the Asia-Pacific area since the late 1990s raising considerable public health concerns (Yip et al., 2013). Two transmembrane proteins, human PSGL-1 (P-selectin glycoprotein ligand-1) and human SCARB2 (scavenger receptor B2), have been identified as the functional receptors for EV71 (Nishimura et al., 2009; Yamayoshi et al., 2009). Unlike human PSGL-1, human SCARB2 is expressed in a wide range of tissues, including neurons in the central nervous system, and functions as a receptor for all tested EV71 strains. More importantly, human SCARB2 not only binds to EV71 but induces EV71 uncoating in a low pH environment (Yamayoshi et al., 2013), which is consistent with the finding that endosomal acidification is essential for EV71 infection (Lin et al., 2012). SCARB2 has a novel fold with a twisted β-barrel core (Neculai et al., 2013). It appears that residues 144–151 of a three-helix bundle at the head region of human SCARB2 are directly involved in EV71 binding according to two independent studies (Yamayoshi and Koike, 2011; Chen et al., 2012). Nevertheless, there is some uncertainty whether there are also other residues involved in virus attachment. Thus the human SCARB2-dependent uncoating of EV71 remains enigmatic.

Dang et al. now present structural and functional studies of human SCARB2 (Dang et al., 2014). Human SCARB2 structures at both neutral and acidic conditions were determined by X-ray crystallography, showing a pH-dependent conformational change of the three-helix bundle. Structural comparisons indicate that the helical bundle acts as a “cap” that regulates the accessibility of the entrance to a large cavity which traverses the whole length of the ectodomain and might be a lipid-transfer tunnel.

Recombinant human SCARB2 was shown to facilitate the expulsion of sphingosine (a pseudo-pocket factor) from EV71 saturated with radioisotope labelled sphingosine under acidic conditions. Furthermore, the transition of native EV71 to A particles on binding SCARB2 demonstrated that EV71 undergoes uncoating upon incubation with human SCARB2 in a low pH environment. *In vitro* binding assays, employing synthetic peptides together with *in silico* docking, suggested the three-helix bundle binds to the EV71 “canyon” and that the entrance of the putative lipid-transfer tunnel is in proximity to the hydrophobic pocket underneath the “canyon” at neutral pH. Taken together, these findings offer a novel model for human SCARB2-dependent cell entry of EV71 in which human SCARB2 serves as a molecular switch that aids in the expulsion of the pocket factor and triggers EV71 uncoating upon acidification.

The expulsion of the labelled sphingosine by binding of SCARB2 supports the suggestion that the receptor binds into the canyon as proposed by Dang et al. Hence, an alternative explanation of the above observations is that binding of SCARB2 expels the pocket factor and destabilizes the virus in much the same way as the other receptor molecules that are known to bind into the canyon of EVs.

Another aspect of the study by Dang et al. is that acid-induced conformational change of the “cap” region might be a common basis for pH-dependent interactions of SCARB2 with its ligands including both EV71 (an exogenous ligand) and β-glucocerebrosidase (β-GC, an endogenous ligand), as SCARB2 was known to deliver β-GC from the endoplasmic reticulum to lysosomes (Zachos et al., 2012). Thus it would be interesting to determine which residues are important for the pH-induced structural changes and whether substitutions of these residues would impair the ability of SCARB2 to trigger EV71 uncoating and transport β-GC. More importantly, SB-RI and CD36, which are homologous to SCARB2, have so far been shown to transport cholesterol and fatty-acids, respectively (Neculai et al., 2013). SCARB2, however, hasn’t been demonstrated to possess the ability of binding and/or delivering lipid despite containing a large cavity that might accommodate lipid ligands. Therefore, the functional importance of this large cavity remains to be further characterized.

Although Dang et al. have made some interesting suggestions regarding the interaction of EV71 with human SCARB2, there are still many other questions that need to be answered. For instance, does human SCARB2 induce the uncoating in other viruses that can interact with SCARB2 such as coxsackievirus A7, A14 and A16 (Yamayoshi et al., 2012)? If yes, would the acid-triggered conformational change be a major factor for the uncoating of these viruses? Is it possible to design agents that interfere with the pH- dependent conformational change of SCARB2 or bind to the presumable lipid-transfer tunnel? Can these agents be selectively designed so as to inhibit EV71 and other related viruses without affecting the normal function of SCARB2 in host cells?

In summary, the work by Dang et al. provides a new model for receptor-mediated entry of picornaviruses into host cells that depends on the pH-triggered conformational change of the receptor molecule. The detailed molecular basis of the interaction between EV71 with human SCARB2 and the functional importance of the large cavity within SCARB2 still remains open questions for future studies.
